# Vertical transport and spatiotemporal dynamics of giant viruses in the North Pacific subtropical gyre

**DOI:** 10.1093/ismejo/wraf094

**Published:** 2025-05-22

**Authors:** Md Moinuddin Sheam, Elaine Luo

**Affiliations:** Department of Biological Sciences, University of North Carolina at Charlotte, Charlotte, NC 28223, United States; Center For Computational Intelligence to Predict Health & Environmental Risks, University of North Carolina at Charlotte, Charlotte, NC 28262, United States; Department of Biological Sciences, University of North Carolina at Charlotte, Charlotte, NC 28223, United States; Center For Computational Intelligence to Predict Health & Environmental Risks, University of North Carolina at Charlotte, Charlotte, NC 28262, United States

**Keywords:** virus, metagenomics, oligotrophic open ocean, biogeochemistry, carbon export, spatiotemporal dynamics

## Abstract

Nucleocytoplasmic large DNA viruses, or “giant viruses,” are prevalent in marine environments, infecting diverse eukaryotic lineages and influencing the marine carbon cycle. Their genomes harbor wide range of auxiliary metabolic genes that influence biogeochemical processes. This study integrates planktonic (5–4000 m) and particle-associated (4000 m) metagenomic samples in the North Pacific Subtropical Gyre, along with particulate export flux data at 4000 m, to investigate the vertical transport of giant viruses and their correlation with carbon export through space and time. By analyzing metagenomic samples over a period of 6 years across 15 depths, we curated a database of 37 giant virus population genomes and 1496 contigs and investigated their spatiotemporal variability and functional capacity in the open ocean. We reported multiple lines of evidence supporting the viral shuttle hypothesis, including the vertical transport of giant viruses from the upper ocean to abyssal depths and their positive correlation with particulate carbon export flux at 4000 m, particularly a giant species closely related to *Phaeocystis globosa* virus known to infect a bloom-forming alga. We identified giant viruses encoding diverse auxilary metabolic genes, including genes associated with photosynthesis, nutrient transport, and energy metabolism. These auxiliary metabolic genes displayed depth-specific distributions, which we postulate reflect depth-specific adaptations to light-energy and nutrient-limited conditions along the water column. This study provides critical insights into biogeochemical impacts of giant viruses by identifying key giant viruses that can impact export processes and depth-specific distributions of auxiliary metabolic genes impacting biogeochemical processes along the open ocean water column.

## Introduction

The discovery of Nucleocytoplasmic large DNA viruses (NCLDV), or “giant viruses” of the phylum *Nucleocytoviricota,* led to a paradigm shift in virology by blurring the line between viruses and cellular organisms [1, 2]. This diverse group of eukaryotic viruses features large virions, with sizes reaching up to 1.5 μm and genomes that can extend up to ~2.5 million base pairs [3], comparable to the size of prokaryotic cells. The first giant virus (*Acanthamoeba polyphaga* mimivirus) was discovered in 2003, and since then, several culture-based approaches have identified giant viruses infecting Amoebazoa [4]. In recent years, metagenomic analysis complemented the culture-based approaches to understand the diversity and ecological significance of these viruses. The first giant virus metagenome-assembled genome (GVMAG) was reconstructed in 2011 [5]. Large-scale metagenomic-based approaches have greatly expanded our understanding of giant viruses, especially by reconstructing 2074 GVMAGs [6] and 501 GVMAGs primarily from diverse environments [1].

Giant viruses are globally distributed in different ecosystems and they are particularly diverse and abundant in marine environments [7]. Field studies estimated that there are 10^4^–10^6^ giant viruses per milliliter of seawater [8]. In the marine environment, giant viruses infect a broad spectrum of eukaryotic lineages including haptophytes, choanoflagellates, and dinoflagellates [9–11]. *Nucleocytoviricota* is monophyletic and have been partitioned into six taxonomic orders [12]. Of these, *Imitevirales, Algavirales, and Pandoravirales* primarily infect a wide variety of algae and heterotrophic protists, while *Asfuvirales*, *Chitovirales*, and *Pimascovirales* are associated with infections in various protists and metazoan hosts [7]. By infecting a diverse range of eukaryotic species, giant viruses play important role for regulating microeukaryotic community structure in the ocean [13].

The genome of giant viruses encodes proteins that can influence the biogeochemical processes. Metagenomic studies and isolated giant viruses revealed that their genomes are complex and chimeric, suggesting they acquired numerous genes from a diverse range of cellular lineages and other viruses [14–16]. These viruses contain proteins typically considered as signatures of cellular life [17]. Giant viruses encode proteins associated with the carbon metabolism, light harvesting and energy metabolism [18]. Different categories of auxiliary metabolic genes (AMGs) associated with glycolysis, TCA cycle, and fermentation are broadly distributed in the members of giant viruses [19]. Giant viruses also contain proteins with a putative role in photosynthesis and different substrate transport processes [6]. These AMGs underscore the potential impact of these viruses on biogeochemical processes in the marine environment by modulating nutrient transport, photosynthesis, and carbon metabolism. The environmental distribution of giant virus-encoded AMGs along the open ocean water column, which can reveal their depth-specific adaptations, remains open to investigation.

Viruses play important role in carbon cycling in the ocean [20]. Identifying whether viruses influence oceanic carbon cycling through their export from shallower water to abyssal depth on sinking particles relates to the “viral shuttle” hypothesis [21–23], a conceptual model that proposes viral lysis can enhance carbon export to the deep sea. In the viral shuttle model, viral lysis of cells releases sticky substances, leading to increased aggregate formation, biomass sinking, and carbon export from surface ocean to the deep sea [24]. Though evidence supporting the viral shuttle hypothesis was initially based on laboratory observations of viruses infecting phytoplankton [24–26], other reports in the North Atlantic indicated that this process may be generalizable to phytoplankton in the field. Giant coccolithovirus infection of the bloom-causing photosynthetic eukaryotes Emiliania huxleyi in the North Atlantic were associated with particle aggregation and greater downward vertical carbon flux [27, 28]. Further investigation in prokaryote-dominated open ocean indicated that the viral shuttle hypothesis may be generalizable across domains. Viruses infecting photoautotrophic prokaryotes were found to be positively correlated with modelled carbon export flux [29], suggesting that viruses infecting smaller primary producers could enhance export to the deep sea. Viruses infecting heterotrophic bacteria were positively correlated with carbon export flux to abyssal depths [30], suggesting that viruses targeting particle-degrading bacteria can enhance carbon export efficiency. Whether any of these processes relating to the viral shuttle hypothesis is generalizable to giant viruses, however, remains open to investigation.

Despite their potential to influence marine biogeochemistry, giant viruses remain understudied compared to other viruses in the ocean, due to large capsid sizes that preclude their detection in viral metagenomic studies focused on particles passing through 0.2 μm filters. For example, recent studies have highlighted the rich diversity (>16 000 viral population genomes) of bacteriophages found along the water column and on sinking particles at Station ALOHA located in the North Pacific Subtropical Gyre [30–32], but only 11 giant virus metagenome-assembled genomes have been reported in this environment [33]. Furthermore, no study to date has simultaneously analyzed planktonic and sediment trap samples to investigate the role of giant viruses on particulate carbon export *in situ*.

In this study, we used both planktonic and sediment trap samples to identify the vertical transport of giant viruses from the surface ocean to the deep sea, as well as their correlation with particulate carbon export flux. We analyzed 798 planktonic metagenomic samples, largely from the >0.2 μm size fraction, collected over a total period of six years from depths ranging from 5 m to 4000 m, along with 63 sediment trap samples from abyssal depths (4000 m) collected over three years overlapping with the planktonic dataset. We identified 468 giant virus populations that were vertically transported from the water column to abyssal depths. Moreover, 101 giant virus populations were positively associated with particulate carbon export flux at 4000 m. A diverse range of AMGs associated with nutrient transport, carbon and nitrogen metabolism were identified, revealing distinct depth distributions that we postulate reflect depth-specific adaptations to light-energy and nutrient adaptation along the water column. Our study suggested the vertical transport of giant viruses from the surface to the abyssal ocean on sinking particles and their positive correlation with *in situ* particulate carbon export flux. We also identified depth-dependent distribution of diverse giant virus encoded auxiliary metabolic genes as well as distinct spatiotemporal distribution of these viruses in the North Pacific Subtropical Gyre.

## Materials and methods

### Metagenomes used and bioinformatic workflow

We analyzed three planktonic metagenomic datasets derived from previous studies conducted at Station ALOHA (22°45′ N, 158° W), located in the Pacific Ocean ~100 km north of Hawai'i, and collected on Hawai'i Ocean Time-series cruises at approximately monthly intervals. The first dataset (ALOHA 1.0) contains 107 metagenomes collected from 2010-2011 from 25 - 1000 m [34]. The second dataset (ALOHA 2.0) consists of 691 metagenomes from 5 - 4000 m between 2014 and 2017 [32, 35]. Briefly, seawater filtered through 1.6 μm and 0.2 μm filters for the ALOHA 1.0 dataset, and through 0.2 μm and 0.02 μm filters for ALOHA 2.0 dataset, followed by DNA extraction from filters and sequencing on the MiSeq and NextSeq 500 platforms (Illumina). In addition to these two pelagic datasets, we also incorporated 63 metagenomic samples previously collected from sinking particulate organic matter in a deep-moored sediment trap at 4000 m from Station ALOHA over a period of three years (2014–2016) [30, 36]. These 63 sediment trap samples were used to generate particulate carbon flux data. Briefly, sinking particles were collected in a formalin brine solution to analyze particulate carbon as previously described [36, 37]. Samples that demonstrated ≥ 150% of the 28-year mean carbon flux were identified summer export pulse samples [30]. Metadata for these datasets are available on Table S1. Technical details on the bioinformatic workflow can be found in supplementary methods and Fig. S1.

## Results and discussion

### Distribution of giant viruses across depth

**Figure 1:**
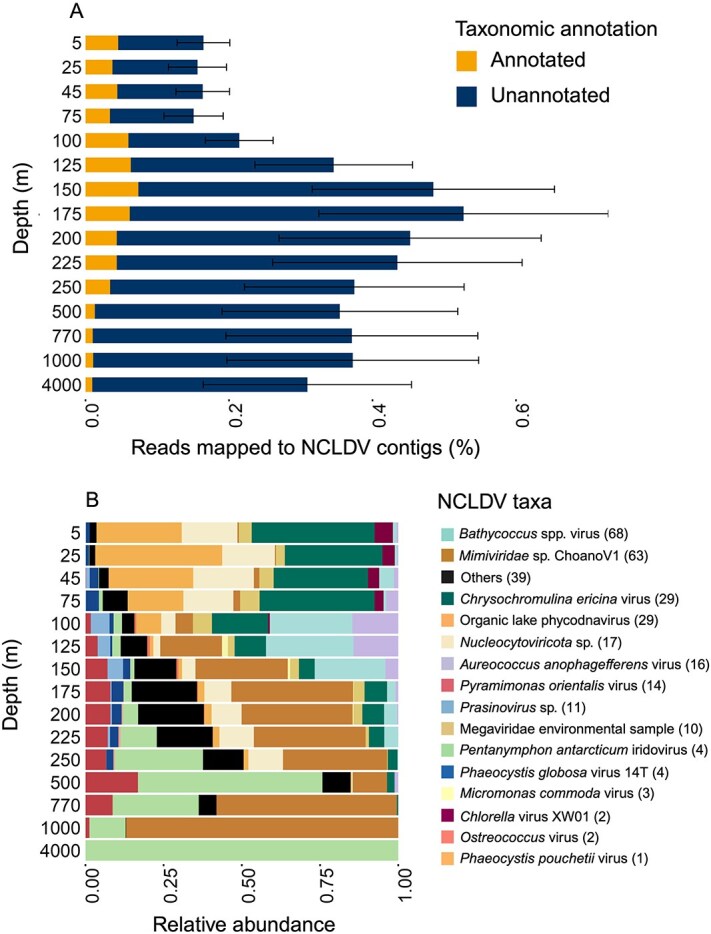
(A) Percentage of read mapped (mean ± SE) to taxonomically identified and novel NCLDV contigs across depths. (B) Relative abundance of viruses closely-related to NCLDV species across the water column (0005 m–4000 m). Relative abundance was calculated using Q2Q3 coverage normalized to the total of 1. Legend shows the number of contigs classified in each NCLDV taxa.

Based on the presence of NCLDV-specific marker genes, we assigned species-level taxonomy to 20.9% of the 1496 NCLDV contigs. Among these, prasinoviruses are the most taxonomically identified group which include 66 contigs closely related to *Bathycoccus* sp. RCC716 virus, 11 contigs closely related to *Prasinovirus* sp. and two contigs closely related to *Bathycoccus* sp. *RCC1105* virus. We also identified giant virus contigs closely related to *Mimiviridae* sp. ChoanoV1 (n = 64) and *Chrysochromulina ericina* virus (n = 29) (Fig. 1B, Table S2). However, we also found 36 NCLDV contigs that were closely related to mammalian poxvirus (Table S2), which can be due to shared conserved sequences or limited representation of closely related giant viruses in the database.

Around 0.5% of metagenomic reads across 15 depths were mapped to NCLDV contigs, with a peak in read recruitment between 150 m and 225 m (Fig. 1A). Within taxonomically identifiable NCLDV contigs, viruses from the *Mesomimiviridae* family were also numerous (n = 62). These viruses include putative Organic lake phycodnavirus (n = 29*),* putative *Chrysochromulina ericina* viruses (n = 29*), and* putative *Phaeocystis globosa* viruses 14T (n = 4*).* Putative Organic lake phycodnaviruses *and Chrysochromulina ericina* viruses were relatively abundant in the upper epipelagic zone (5 m to 75 m). Photosynthetic haptophytes, such as *Chrysochromulina ericina* [38], are widespread throughout the euphotic zone of the world's oceans [39] and are known to serve as hosts NCLDVs [38]. At Station ALOHA, the relative abundance of putative *Chrysochromulina ericina* viruses and closely related Organic lake phycodnavirus was abundant in surface waters and decreased sharply at the deep chlorophyll maximum (DCM) at 100 m to 125 m (Fig. 1B), consistent with the expectation that they target photosynthetic haptophytes in the photic ocean. In contrast, giant virus species closely related to *Bathycoccus* sp. RCC716 virus peaked at the DCM, an observation consistent with previous observations in that the green algae *Bathycoccus* is prevalent primarily at the DCM in tropical ocean waters [40].

**Figure 2:**
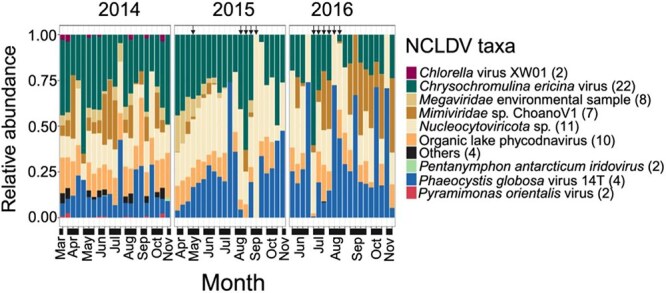
Relative abundance of viruses closely-related to NCLDV species in the sediment trap samples across three years (2014–2016). Relative abundance was calculated using Q2Q3 coverage normalized to max of the sample. One sample (SRR7648310) was excluded from visualization since it does not contain any taxonomically identified NCLDV species top arrows indicate the sample with summer export pulse.

Giant viruses that were identified as *Mimiviridae* sp. ChoanoV1 displayed increased relative abundances below the DCM (125 m), peaking in deeper mesopelagic zones (770 m and 1000 m). This observation is consistent with a previous report showing that *Mimiviridae* sp. ChoanoV1 infects choanoflagellate [11] abundant in the dark ocean [41]. Putative *Pentanymphon antarcticum iridovirus* showed an increase in relative abundances below 225 m, peaking at 500 m, and was the only taxonomically identified giant virus species detected at the 4000 m water column. This virus was recently identified from the Antarctic seaspider *P. antarcticum* collected at a depth of 870 m [42]. Our study highlights distinct distribution patterns among giant viruses along the open ocean water column, likely driven by the distribution of their hosts.

### Evidence for vertical transport of giant viruses to abyssal depths

Metagenomic samples from sediment traps collected at 4000 m sinking particles at Station ALOHA (2014–2016) were analyzed to investigate giant viruses associated with sinking particles at abyssal depths. Reads from 63 previously collected sediment trap samples were recruited onto 1496 NCLDV contigs and normalized Q2Q3 coverage was calculated (Table S3). Among the 1496 NCLDV contigs, 468 were detected (non-zero Q2Q3 coverage) in the 4000 m sediment trap samples (Fig. S2). The giant viruses were predominantly novel (~84%), as only 72 out of the 468 contigs identified in the sediment traps could be taxonomically classified. Novel giant viruses in the sediment traps might infect deep-sea grazers. The majority of the taxonomically identified contigs were closely related to *Chrysochromulina ericina* virus (n = 22), followed by *Nucleocytoviricota* sp. (n = 11) and Organic lake phycodnavirus (n = 10).

Several NCLDV species were consistently detected in sinking particles collected at 4000 m over three consecutive years. Viruses identified as *Chrysochromulina ericina* virus showed a high relative abundance in the upper surface layers (5 m to 75 m) and was almost consistently present in sediment trap samples throughout three years (Fig. 1B, Fig. 2). *Chrysochromulina ericina* virus infects haptophyte *Chrysochromulina ericina*, that can occasionally form bloom [43]. Putative Organic lake phycodnavirus originated in the upper surface of the ocean, were vertically transported to sediment and showed consistent presence in the sediment trap samples. This suggests that these viruses originated in the 5 m and transported vertically to the 4000 m sediment via sinking particles. In the mesopelagic region, viruses closely related to *Mimiviridae* sp. ChoanoV1 was relatively abundant and demonstrated the presence in the sinking particle-associated sediment-trap samples at 4000 m, particularly with higher abundance in 2016 (Fig. 2).

Giant virus species closely related to *P. globosa* virus 14T was found in very low abundances in 5 m to 250 m planktonic samples (Fig. 1B), but dominated 4000 m sediment trap samples. In several sediment trap samples collected from 2015 and 2016, *P. globosa* virus accounted for ~75% of the relative abundance among the taxonomically identified NCLDV species (Fig. 2). *P. globosa* virus 14T can potentially infect *P. globosa*, a known bloom-causing species the coastal [44, 45], north temperate, tropical, and subtropical ocean [46]. A previous study from the southern North Sea found that during a *P. globosa* bloom, there was a significant increase in the abundance of *P. globosa* viruses, suggesting that they contribute to the mortality of this bloom-forming alga [47].

Several species exhibited high relative abundance in the water column but were either absent or present in very low abundance in the sediment trap. For instance, viruses closely related to *P. antarcticum iridovirus* was relatively abundant at greater depths, especially at 500 m and 4000 m, but it was rarely detected in the sediment trap, appearing in only four samples. Additionally, viruses closely related to *Aureococcus anophagefferens* virus, *Bathycoccus* sp. RCC716 virus, and *Prasinovirus* sp. were prevalent at the DCM, yet these species were not found in the 4000 m sediment-trap samples (Fig. 1B, Fig. 2). These findings indicate these giant virus taxa are not associated with the vertical transport from water column to the abyssal depth.

Our findings are consistent with the viral shuttle hypothesis in that (i) planktonic giant viruses were transported to deep sea on sinking particles, (ii) the vertical transport of giant viruses is likely an active (e.g. lysis and aggregation) and not passive (e.g. adsorption) process, as the most abundant giant viruses detected at 4000 m sediment traps were not abundant in the water column and vice versa, and (iii) the most abundant identifiable giant virus detected at 4000 m sediment traps are closely related to giant virus species known to infect bloom-causing phototroph, *P. globosa and Chrysochromulina ericina* virus. Overall, our study provides strong evidence that several giant virus species were transported via sinking particles to the abyssal ocean at 4000 m.

### Giant virus correlation with particulate carbon export flux

**Figure 3:**
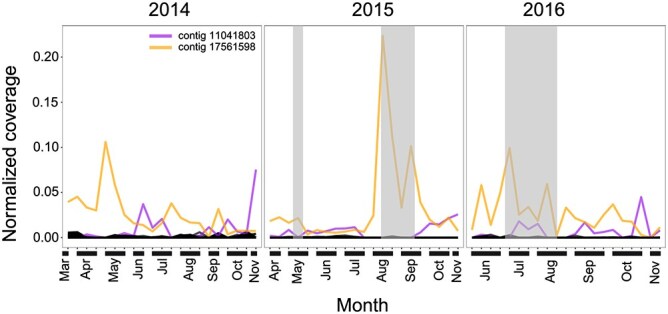
Normalized Q2Q3 coverage of NCLDV contigs in the 4000 m sediment trap samples from 2014 to 2016 that exhibited a significant positive correlation with particulate carbon export flux. The Q2Q3 coverage was normalized to the sum total of 1 in a sample. Highlighted samples indicate the summer export pulse, during which carbon flux was ≥150% of the 28-year mean. NCLDV contigs with visibly higher coverages during heightened carbon export (shaded area of graph) are shown in color.

Weighted gene co-expression network analysis (WGCNA) identified 101 NCLDV contigs that were significantly (*P* < .05) positively correlated with particulate carbon export flux (Fig. 3). Taxonomic annotation of 35 of these contigs revealed several NCLDV species closely related to *Chrysochromulina ericina* virus (n = 11), Organic lake phycodnavirus (n = 5), *Chlorella* virus XW01 (n = 2), *P. globosa* virus 14T (n = 2), *Pyramimonas orientalis* virus (n = 2), and *Mimiviridae* sp. ChoanoV1 (n = 1) demonstrated positive correlation with particulate carbon export flux. Contigs that were positively correlated with carbon export through WGCNA analysis were also enriched during the summer export pulse (Fig. 3).

**Figure 4:**
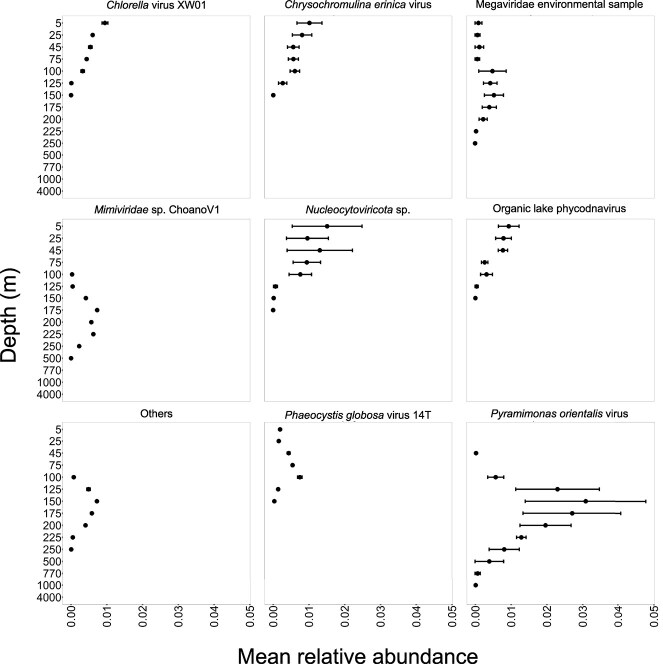
Depth of origins of viruses closely-related to NCLDV species associated with particulate carbon export flux. Solid circle shows the relative abundance calculated using Q2Q3 coverage normalized to the total of 1 in that sample and averaged through time (mean ± SE).

The majority of the NCLDV contigs positively correlated with particulate carbon export flux were observed only in the upper ocean (5 m to 250 m) (Fig. 4A-C, 4E-I, Table S4). Only a single contig closely related to *Mimiviridae* sp. ChoanoV1 showed a positive correlation with carbon export, which was first detected at 225 m, with its highest abundance observed at 1000 m (Fig. 4D). In contrast, viruses closely related to *Chlorella* virus XW01, *Chrysochromulina ericina* virus, Organic lake phycodnavirus, *P. globosa* virus 14T associated with carbon export were predominantly observed in shallower water (<100 m) (Fig. 4). Our findings reveal that giant viruses that were positively correlated with particulate carbon export flux mainly originated in the photic zone and were transported to the deep sea. Previous study on prokaryotic viruses from Station ALOHA found 194 viral populations were positively correlated with particulate carbon export flux to the abyssal ocean [30]. In terms of giant viruses, the composition of the eukaryotic virus community can predict carbon export efficiency in the marine environment [48]. Giant virus infection of the *Emiliania huxleyi* bloom was associated with particle aggregation and greater downward carbon flux in the North Atlantic [27, 28]. Together, this study provides multiple lines of evidence that is consistent with the idea that virus-mediated processes can facilitate particle export to the deep sea, and expand our understanding of the viral shuttle hypothesis to include giant viruses in the open ocean.

### Environmental distribution of giant virus AMGs

Giant viruses contain different AMGs that can manipulate the physiology of the host and facilitate viral propagation [7, 18, 19]. Investigating the environmental distribution of giant virus-encoded metabolic genes can reveal depth-specific adaptations to energy and nutrient limitations in the oligotrophic ocean.

**Figure 5:**
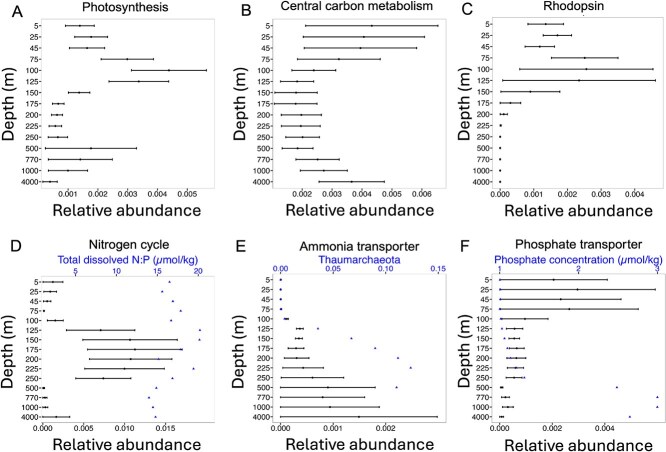
Solid circles demonstrate depth profiles, averaged through time (mean ± SE), of the abundance-normalized proportion of giant viruses containing different AMGs: (A) photosynthesis, (B) central carbon metabolism, (C) rhodopsin, (D) nitrogen cycle (solid circle) and ratio of total dissolved nitrogen and phosphorus (blue triangle) (E) sodium dependent ammonium transporter (solid circle) and concentration of Thaumarchaeota (syn. Nitrososphaerota) shown as blue triangle, (F) phosphate transporter (solid circle) and phosphate concentration (blue triangle). Environmental metadata were retrieved from previous studies at station ALOHA [32, 34] and Hawaii Ocean Time-series HOT-DOGs application.

We identified giant viruses carrying genes associated with photosynthesis, including photosystem components, ferredoxin, and chlorophyll a/b binding protein. The relative abundances of giant viruses with photosynthesis genes increased over threefold from surface waters to the DCM (Fig. 5A). Genes associated with light-energy generation are widespread in giant viruses to facilitate virion production [6]. In response to viral infection, protists suppress their photosynthetic light-harvest capacity and the presence of light-harvesting genes in the giant viruses can increase photosynthetic capacity of the virocell [1]. We observed a three-fold increase in the relative abundances of giant viruses carrying photosynthetic genes at the low-light depths near the DCM, relative to the surface ocean, which is consistent with the previous study on bacteriophage assemblages from Station ALOHA [30]. Taken together, both *in situ* studies reveal potential convergent evolution that present new perspectives to previous lab-based studies focused on cyanophage infecting high-light cyanobacteria, which suggested that virus-encoded photosystem genes help hosts overcome photoinhibition and are useful in high light environments [49–51]. We postulate that giant virus-encoded photosynthesis genes can be advantageous in the low-light conditions to help hosts overcome light-energy limitation, and that these genes can reflect giant virus adaptation to low-light environments.

Giant viruses from Station ALOHA contained genes for central carbon metabolism processes including glycolysis, TCA cycle, and pentose-phosphate pathway. Giant viruses with central carbon metabolism genes were more abundant in the upper surface layers (5 m to 45 m) and declined around the DCM. (Fig. 5B). Previous studies have reported the presence of different central carbon metabolism genes in the members of *Nucleocytoviticota* [33]. Manipulation of central carbon metabolism processes is a widespread strategy performed by many giant viruses and frequently observed during the viral infection in oceanic surface water [52]. However, the relative abundance of giant viruses carrying these genes increased again at deeper mesopelagic and abyssal depths (770 m to 4000 m), suggesting that central carbon metabolism genes may be adaptive also at deeper depths in the open ocean.

Metagenomic analyses have also revealed that giant viruses harbor a diverse array of rhodopsins [6, 11]. The only category of rhodopsins we recovered from giant viruses contigs were sensory rhodopsins, which peaked at the DCM (Fig. 5C). These sensory rhodopsins may complement the host’s rhodopsin system [19], particularly at the light-limited depths of the DCM.

Seven NCLDV contigs were found to carry other genes involved in the nitrogen cycle, such as nitrite reductase and ammonia monooxygenase. Genese associated with nitrogen cycling were previously found in giant viruses [53]. Giant viruses carrying these genes peaked in relative abundance at depths between 125 m and 250 m (Fig. 5D). The presence of these genes may enhance nitrogen metabolism to facilitate viral particle synthesis, which can be particularly advantageous in nitrogen-limited marine ecosystems. Our discovery of depth-specific distributions of key AMGs *in situ* provides insight into viral adaptations to biogeochemical gradients along the open ocean water column.

Giant viruses with ammonium transporter genes gradually increased below 200 m, peaking at 4000 m (Fig. 5E). Of the four giant virus contigs carrying ammonium transporter genes, none were taxonomically unidentifiable through marker gene analysis. Ammonium transporter has been previously found in giant virus infecting the green photosynthetic algae *Ostreococcus* virus and was thought to increase the uptake of the NH4+ to fulfill the increased nitrogen requirement of the infected cell [54], but not yet in giant viruses infecting mixotrophic and heterotrophic protists that we expect dominate the aphotic ocean. Despite observations that ammonia is nearly consistently undetectable and limiting throughout the water column at Station ALOHA [55], the proportion of giant viruses encoding ammonium transporters increased below the photic zone. An explanation could be due to the presence of ammonia-oxidizing Thaumarchaeota (syn. *Nitrososphaerota*), which also increase in abundance below the photic zone [32], have high affinity for ammonia in the marine environment [56], and can outcompete other microbes for ammonia in ammonia-limited environments [57]. A previous study revealed that ammonium transporters encoded by bacteriophages were enriched in the aphotic ocean at depths where Thaumarchaeota (syn. *Nitrososphaerota*) were more abundant, suggesting that bacteriophages might encode copies of ammonium transporter genes to help their bacterial hosts compete with Thaumarchaeota (syn. *Nitrososphaerota*) for a limited resource [32]. Here, we postulate that giant virus-encoded ammonium transporters may also facilitate ammonium acquisition to meet the increased nitrogen demands of infected cells, particularly at depths with Thaumarchaeota (syn. *Nitrososphaerota*) competitors. Taken together, our results show that, despite being two distinct groups of viruses with very different hosts, both bacteriophages (prokaryotic hosts) and giant viruses (eukaryotic hosts) encode ammonium transporter genes, and viruses encoding for these genes from both groups display similar depth distributions at Station ALOHA. This remarkable similarity across distinct virus-host systems may reflect convergent evolution driven by selective pressures from Thaumarchaeota (syn. *Nitrososphaerota*) competition in ammonia-limited environments.

We found giant viruses encoding different transporter genes for nutrient acquisition. In the nutrient-limited oligotrophic ocean, transporter genes were hypothesized to increase the acquisition of essential nutrients during virion production [19]. We identified viruses closely related to *Mimiviridae* sp. ChoanoV1 encoding sodium-dependent phosphate transporter genes that were highly abundant in the upper epipelagic layer, between 5 m and 75 m (Fig. 5F). Phosphorus concentrations are low in the upper water column in the North Pacific ocean [58], and phosphate transporters may enhance phosphorus acquisition during viral infections in phosphate-limited environments. Viruses are more enriched in phosphorus compared to the cells [59], potentially requiring the uptake of additional phosphorus in the phosphorus-limited deep ocean.

### Functional capacity of NCLDV from station ALOHA

**Figure 6:**
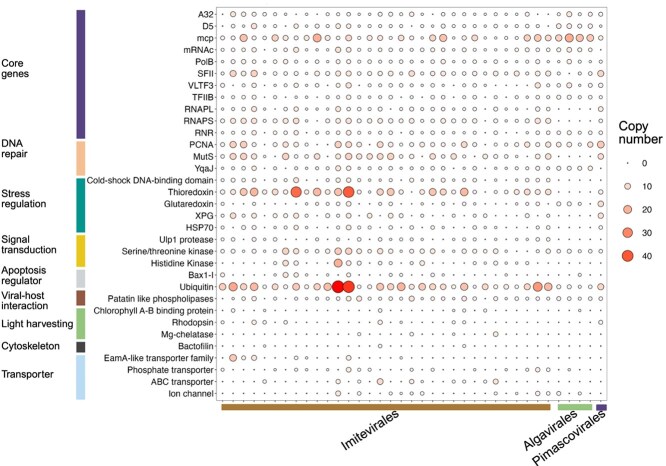
Functional annotation of GVMAGs belonging to three different orders from station ALOHA. The colored bar in the X-axis shows the taxonomic families (orders) of the GVMAGs. The colored bar in the Y-axis represents the functional categories of the genes encoded by the GVMAGs. Size of the circle represents the copy number of genes. Gene abrreviations: A32, DNA packaging ATPase; D5, DNA helicase-primase; mcp – NCLDV major capsid protein; mRNAc, mRNA capping enzyme; PolB, Family B DNA polymerase; SFII, Superfamily II helicase; VLTF3, Late transcription factor 3; TFIIB, Transcription factor IIB; RNAPL, RNA polymerase large subunit; RNAPS, RNA polymerase small subunit; RNR, Ribonucleotide reductase; PCNA, Proliferating cell nuclear antigen (DNA replication clamp); MutS, DNA mismatch repair protein; YqaJ, DNA repair helicase; XPG, Xeroderma Pigmentosum complementation group G; HSP70, Heat shock protein 70; Ulp1 protease, Ubiquitin-like-specific protease 1; Bax1-I, inhibitor of apoptosis-promoting Bax1.

Functional annotation of GVMAGs demonstrated complex genomic repertories. A complete or near-complete set of NCLDV core genes was present in all the GVMAGs, which indicates that these MAGs are of high quality (Fig. 6). These core genes are broadly present in the *Nucleocytoviricota* genomes and are used as phylogenetic markers [7, 60]. None of the four MAGs from the genus *Prasinovirus* (*Algavirales* order) contained a DNA-dependent RNA polymerase (Fig. 6), which is consistent with previous reports [61].

Giant viruses from Station ALOHA were found to carry different stress regulation genes, including thioredoxin and glutaredoxin (Fig. 6, Table S6). The replication of giant viruses inside the host can occur under high oxidative stress conditions. Therefore, the presence of enzymes with oxidative stress regulation capacity can be important for preventing the damage to viral machinery [1]. Thioredoxin is likely to mitigate cellular oxidative stress by neutralizing harmful reactive oxygen species that are produced by the host during viral infection [8] . To maintain high fidelity during genome replication, giant viruses carry genes to repair their own DNA [62, 63]. We found that giant virus genomes contained genes for DNA repair, including those for mismatch repair (mutS) and proliferating cell nuclear antigen (PCNA) and YqaJ-like viral recombinase domain. Homologs of these DNA repair genes are prevalent among giant viruses [64, 65]. However, genomes of *Algavirales* (Prasinoviridae family) did not contain any mutS homologs, which is consistent with the previous findings [52]. Many giant virus genomes have been shown to encode homologs of cytoskeleton components such as actin and myosin [19, 66]. Interestingly, we found the presence of cytoskeletal protein bactofilin in *Imitevirales* giant viruses, which is widespread in bacteria and was not previously reported to present in giant viruses (Fig. 6). We also identified several genes associated with central carbon metabolism (Fig. S3, Table S6), consistent with previous reports of giant viruses encoding metabolic genes associated with energy production, including those involved in glycolysis, the TCA cycle, and the pentose-phosphate pathway [19].

### Phylogenetic diversity of GVMAGs

**Figure 7:**
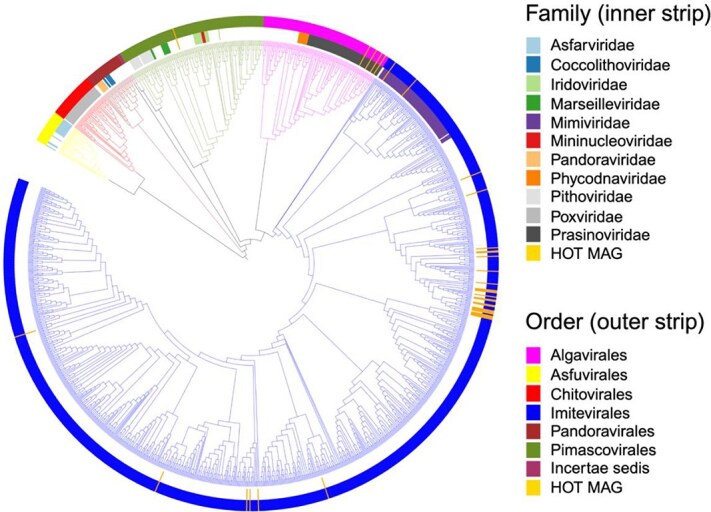
Phylogenetic tree 37 GVMAGs along with 1383 reference genome from the giant virus database. HOT MAG refers to the GVMAGs generated in this study. The tree is rooted at Poxviridae/Asfarviridae branch. The phylogenetic tree was constructed by using highly conserved NCLDV PolB gene obtained from the giant virus database [12] using iTOL [67].

We generated 37 GVMAGs containing at least four out of five NCLDV key marker genes including A32-like ATPase (A32), B-family DNA polymerase (PolB), virus-like transcription factor (VLTF3), NCLDV major capsid protein (MCP), superfamily II helicase (SFII). The size of GVMAGs ranges from 286 to 2263 kbp **(**Fig. S4). Previous study on giant viruses from Station ALOHA constructed 11 GVMAGs with size ranging from ~119–574 kbp [33]. Based on phylogenetic analysis, the majority of these NCLDV MAGs belong to the order *Imitevirales* (n = 32), followed by *Algavirales* (n = 4) and *Pimascovirales (*n = 1) (Fig. 7). All the four GVMAGs from the order *Algavirales* were classified to the genus *Prasinovirus* (Fig. 7, Table S5). Giant viruses from the *Prasinovirus* genus are known to infect prasinophytes (picoeukaryotic algae) including *Bathycoccus*, *Micromonas*, *Ostreococcus,* and *Mantoniella* [68, 69]. *Bathycoccus* sp. RCC716 virus accounted for 21% of the taxonomically identified NCLDV sequences at the contig level, suggesting that GVMAGs from the genus *Prasinovirus* may predominantly infect *Bathycoccus* species at Station ALOHA (Fig. 1B). Six *Imitevirales* MAGs belong to the IM_1 (Table S5), which corresponds to the proposed *Mesomimiviridae* family [52]. Members of *Mesomimiviridae* family are widespread in marine and freshwater ecosystems [12]. Cultivated virus from the *Mesomimiviridae* family infect haptophytes belonging to the genera *Chrysochromulina* and *Phaeocystis* [38]. Our taxonomic identification based on the phylogenetic analysis is also consistent with the TIGTOG [70] (Table S5).

Most *Imitevirales* MAGs (n = 22) belong to the IM_9 family, which includes *Aureococcus anophagefferens* virus (AaV) and *Prymnesium kappa* virus. AaV, the smallest *Imitevirales* member, infects the microalga *Aureococcus anophagefferens,* known for causing harmful algal blooms [71, 72]. Three *Imitevirales* MAGs were classified within the protist-infecting *Mimiviridae* family. We also identified one MAG from the *Pimascovirales* order, which is not a widespread group of viruses in the marine environment [33].

### Spatiotemporal distribution of GVMAGs

**Figure 8:**
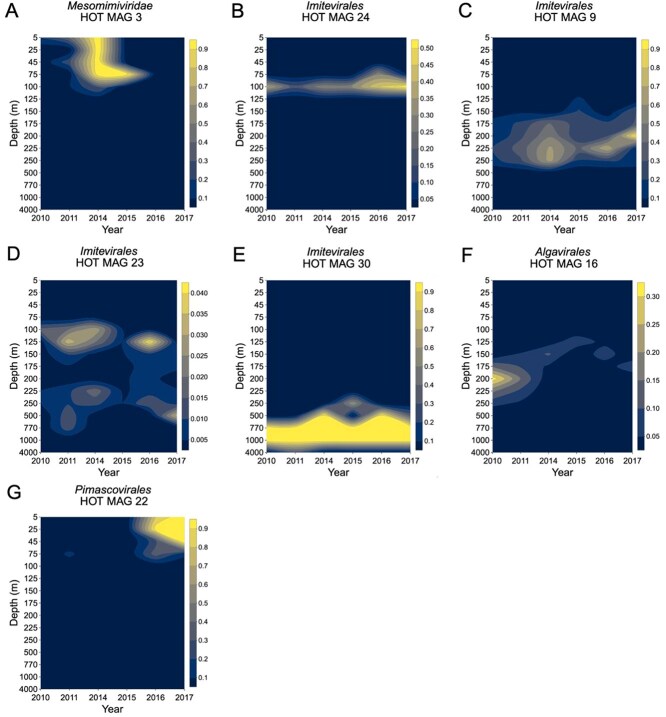
Spatiotemporal distribution of giant virus MAGs from station ALOHA across 15 depths ranging from 5 m to 4000 m over a period of 6 years between 2010–2017. Legend indicates the relative abundance. Relative abundance of each MAG was calculated using Q2Q3 coverage normalized to the maximum coverage that sample. HOT MAG (Hawaii Ocean Time-series metagenome-assembled genome) refers to the of giant virus MAGs generated in this study.

We examined the spatiotemporal distribution of 37 GVMAGs over a period of 6 years across 15 depths. Members of the *Mesomimiviridae* family (belonging to the *Imitevirales* order) are mainly abundant in the surface water (Fig. 8A, Fig. S5), which is consistent with the previous study on giant viruses from Station ALOHA [33]. The *Mesomimiviridae* family is widely distributed in marine environments and its cultivated representatives are known to infect oceanic haptophytes, a group of photosynthetic algae highly abundant in the euphotic zone [8, 39]. Other members of the *Imitevirales* were detected throughout the open ocean water column in this study. One *Imitevirales* GVMAG consistently relatively abundant at the DCM over six years (Fig. 8B), as well as in two years below the DCM, extending to depths of 770 m (Fig. 8C, 8D). One *Imitevirales* GVMAG was highly abundant in the deeper mesopelagic zone, between 770 m and 1000 m over six years (Fig. 8E). Previous studies have also reported the presence of giant viruses from the *Imitevirales* order in deeper pelagic waters. For instance, *Imitevirales* were identified below 200 m and they were abundant at the DCM [8] and in the mesopelagic zone near 500 m at Station ALOHA [33].

*Algavirales* giant viruses were prevalent from the DCM to 770 m (Fig. 8F, Fig. S5). All the MAGs from *Algavirales* order were specifically classified within the family Prasinoviridae. Prasinoviruses are prevalent in oligotrophic waters and known to infect widely distributed prasinophytes in the ocean [8, 73]. Previous findings on giant viruses from Station ALOHA reported the Prasinoviridae family was abundant at depths ranging from 100 m to 500 m [33]. In contrast, the only MAG from the *Pimascovirales* order from this study was prevalent in the shallow depths (<75 m) particularly between 2016 to 2017 (Fig. 8G), consistent with the previous finding from Station ALOHA [33].

## Conclusion

Viruses play significant roles in the biogeochemical cycle in the ocean. To investigate the vertical transport, correlation with carbon export flux and spatiotemporal distribution, we curated a database of 37 giant virus population genomes and 1496 contigs from Station ALOHA. We found 468 giant virus populations were vertically transported from the upper water column to 4000 m. The relative abundance of 101 giant virus populations present in the sediment-trap samples at 4000 m displayed significant positive correlations with particulate carbon export flux. The majority of the giant virus populations associated with sinking particles exported to 4000 m originated from the upper ocean (5–250 m). Together, our study provides multiple lines of evidence consistent with the predicted outcomes of the viral shuttle hypothesis. Furthermore, the environmental distribution of giant virus carrying AMGs showed depth-specific patterns, reflecting potential convergent evolution in viral adaptation to light-energy and/or nutrient limitation across biologically-distinct virus and host systems [32]. For instance, giant viruses with photosynthesis related genes peaked in relative abundance at the low-light depths near the DCM; giant virus carrying phosphate transporters peaked in relative abundance in phosphate-limited depths; and giant virus-encoded ammonium transporters genes, which facilitates ammonia acquisition, peaked in relative abundance at depths with ammonia-oxidizing Thaumarchaeota (syn. *Nitrososphaerota*) competitors. Spatiotemporal distribution of 37 GVMAGs revealed that *Algavirales* were prevalent from the DCM to 770 m, while *Imitevirales* were found from the surface to 1000 m depths. Taken together, our study provides new insights into the vertical transport of giant viruses and their role in carbon export, expanding the relevance of the viral shuttle hypothesis to giant viruses in the open ocean. Furthermore, we demonstrated that giant virus-encoded AMGs potentially drive viral adaptation to low-light or nutrient-limited conditions in the open ocean.

## Supplementary Material

supplimentary_figures_and_methods_wraf094

supplimentary_table_1_wraf094

Supplimentary_table_2_wraf094

Supplimentary_table_3_wraf094

Supplimentary_table_4_wraf094

Supplimentary_table_5_wraf094

supplimentary_table_6_wraf094

## Data Availability

Giant virus assemblies and MAGs are available in figshare (doi:https://doi.org/10.6084/m9.figshare.28734989.v1).
