# Correction to: Vertical transport and spatiotemporal dynamics of giant viruses in the North Pacific subtropical gyre

**DOI:** 10.1093/ismejo/wraf217

**Published:** 2025-10-30

This is a correction to:

Md Moinuddin Sheam, Elaine Luo, Vertical transport and spatiotemporal dynamics of giant viruses in the North Pacific subtropical gyre, *The ISME Journal*, Volume 19, Issue 1, January 2025, wraf094, https://doi.org/10.1093/ismejo/wraf094

In the originally published version of this manuscript, Figure 5 featured the incorrect panel for graph C

This error has been corrected

