# Supplementary material for: Vertical transport and spatiotemporal dynamics of giant viruses in the North Pacific subtropical gyre
Source: ISME J. 2025 May 22;19(1):wraf094. doi: 10.1093/ismejo/wraf094 (PMC12192438; doi:10.1093/ismejo/wraf094)
Supplement: supplimentary_figures_and_methods_wraf094 [file supplimentary_figures_and_methods_wraf094.pdf]

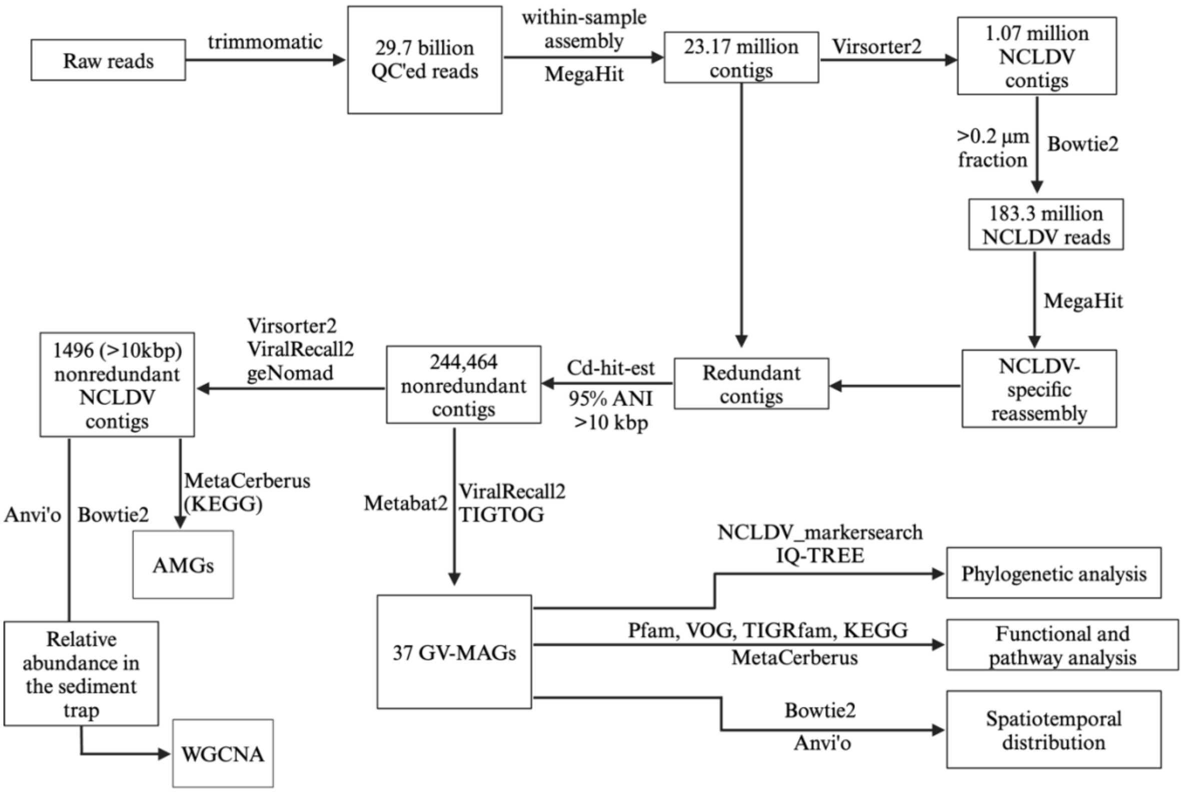

Fig S1: Schematic workflow from metagenomic reads to NCLDV contigs and Giant Virus Metagenome-Assembled Genomes, and analysis

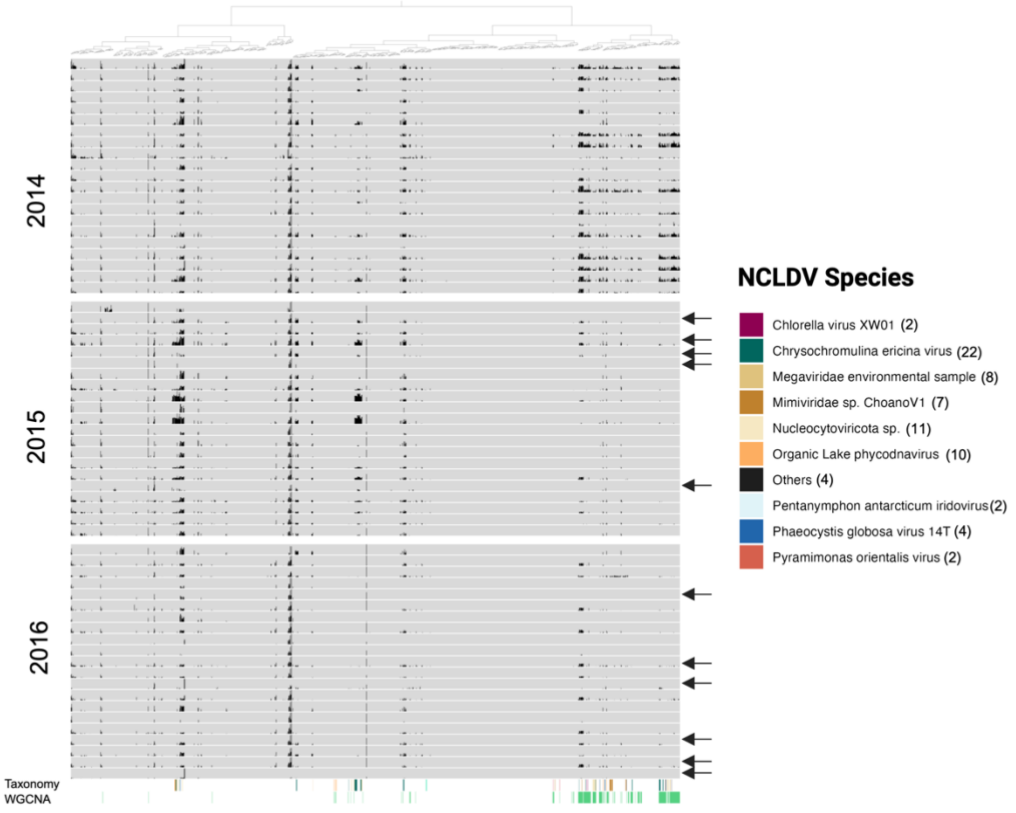

Fig S2: Normalized abundance of NCLDV contigs recovered from planktonic samples on sinking particles collected from 4,000-meter sediment traps. The top dendrogram and associated columns represent the Q2Q3 coverage of individual NCLDV contigs. Each row corresponds to a sample, ordered chronologically from 2014 to 2016. The height of the black bars indicates the Q2Q3 coverage of each contig, normalized to the max of that sample . Contigs with significant correlation to carbon export, as identified by WGCNA, are highlighted in color at the bottom of the figure. The legend shows the number of contigs classified in each NCLDV species. Right arrows show summer export pulse samples based on particulate carbon export flux.

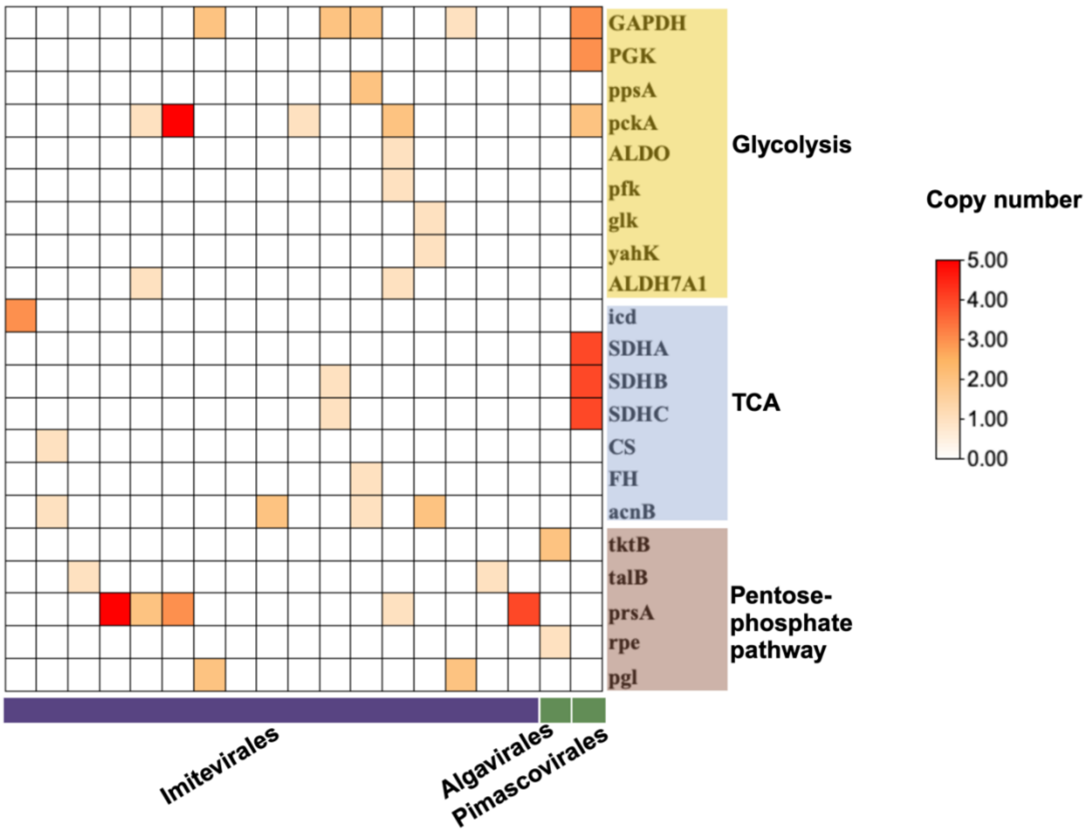

Fig S3: Distribution of central carbon metabolism associated genes in GVMAGs. *The colored bar in the X-axis shows the taxonomic families (orders) of the GVMAGs. Gene abbreviatios: GAPDH – Glyceraldehyde-3-phosphate dehydrogenase; PGK – Phosphoglycerate kinase; ppsA – Phosphoenolpyruvate synthase; pckA – Phosphoenolpyruvate carboxykinase; ALDO – Aldolase (fructose-bisphosphate aldolase); pfk – Phosphofructokinase; glk – Glucokinase; yahK – Putative oxidoreductase; ALDH7A1 – Aldehyde dehydrogenase 7 family member A1; icd – Isocitrate dehydrogenase; SDHA – Succinate dehydrogenase flavoprotein subunit A; SDHB – Succinate dehydrogenase iron-sulfur subunit B; SDHC – Succinate dehydrogenase cytochrome b subunit C; CS – Citrate synthase; FH – Fumarase (fumarate hydratase); acnB – Aconitase B; tktB – Transketolase B; talB – Transaldolase B; prsA – Ribose-phosphate pyrophosphokinase; rpe – Ribulose-phosphate epimerase; pgl – 6-Phosphogluconolactonase*

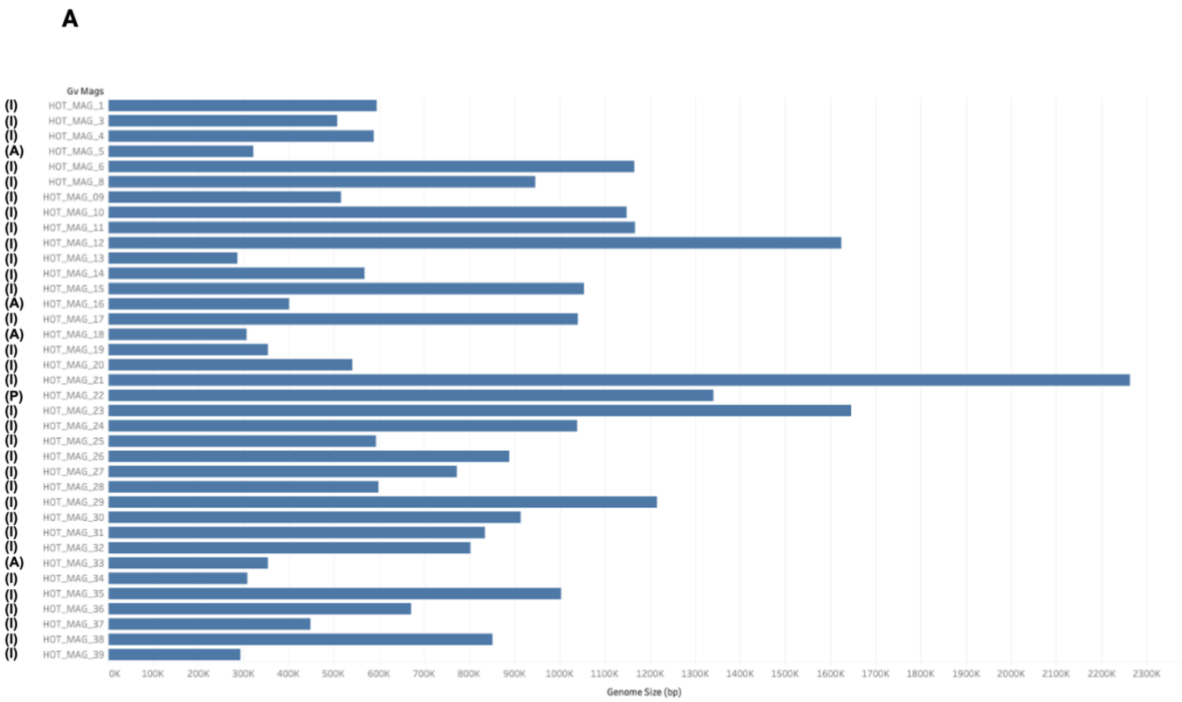
A
Gv Mags
(I) HOT_MAG_1
(I) HOT_MAG_3
(I) HOT_MAG_4
(A) HOT_MAG_5
(I) HOT_MAG_6
(I) HOT_MAG_8
(I) HOT_MAG_09
(I) HOT_MAG_10
(I) HOT_MAG_11
(I) HOT_MAG_12
(I) HOT_MAG_13
(I) HOT_MAG_14
(I) HOT_MAG_15
(A) HOT_MAG_16
(I) HOT_MAG_17
(A) HOT_MAG_18
(I) HOT_MAG_19
(I) HOT_MAG_20
(I) HOT_MAG_21
(P) HOT_MAG_22
(I) HOT_MAG_23
(I) HOT_MAG_24
(I) HOT_MAG_25
(I) HOT_MAG_26
(I) HOT_MAG_27
(I) HOT_MAG_28
(I) HOT_MAG_29
(I) HOT_MAG_30
(I) HOT_MAG_31
(I) HOT_MAG_32
(A) HOT_MAG_33
(I) HOT_MAG_34
(I) HOT_MAG_35
(I) HOT_MAG_36
(I) HOT_MAG_37
(I) HOT_MAG_38
(I) HOT_MAG_39
0K 100K 200K 300K 400K 500K 600K 700K 800K 900K 1000K 1100K 1200K 1300K 1400K 1500K 1600K 1700K 1800K 1900K 2000K 2100K 2200K 2300K
Genome Size (bp)

B

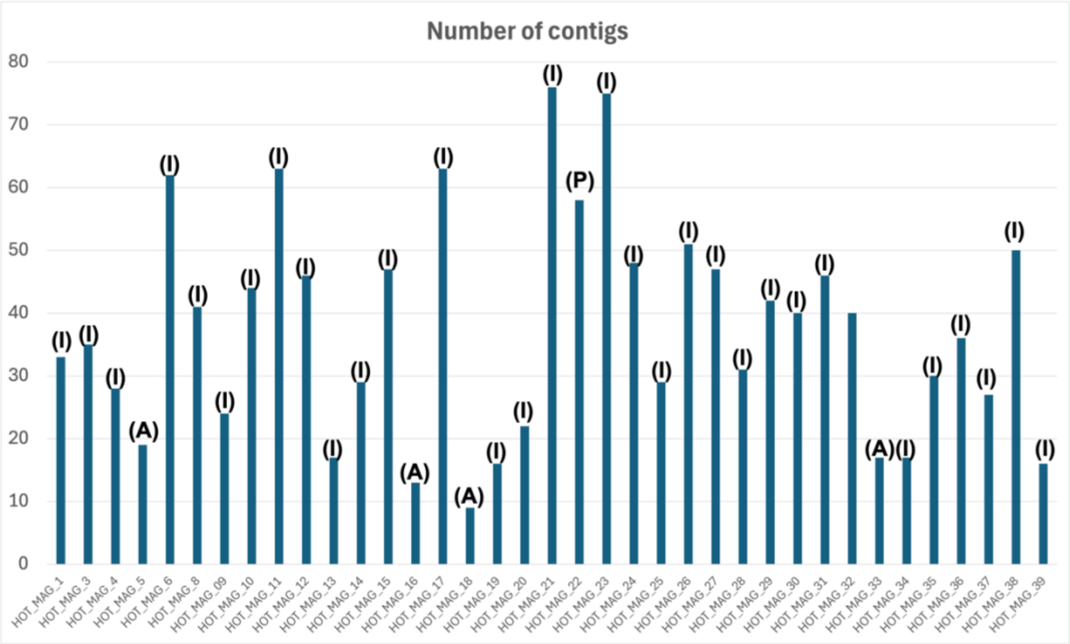

C

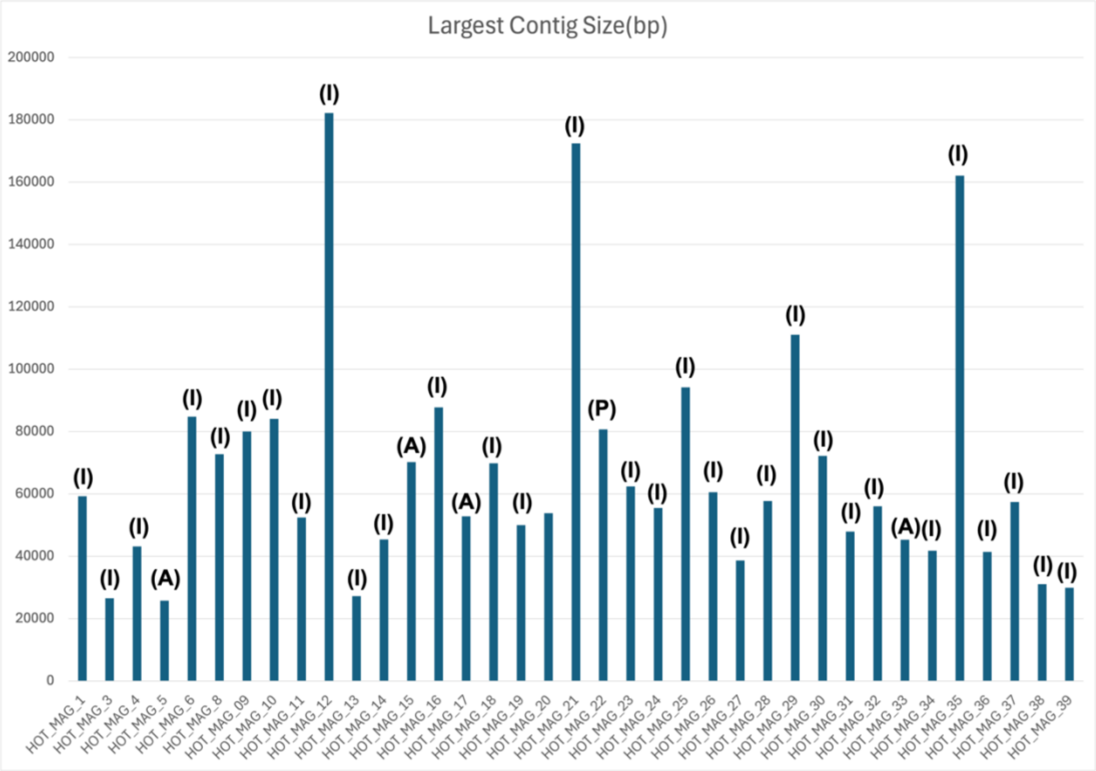

Fig S4: Genome statistics of Giant Virus Metagenome-Assembled Genomes (GVMAGs). (A) Size (kbp) of the GVMAG; (B) Number of contigs in the GVMAG; (C) Size of the largest contigs (bp) in GVMAG. The letter indicates the order of giant virus. A: Algavirales, I: Imitevirales, P: Pimascovirales.

Imitevirales

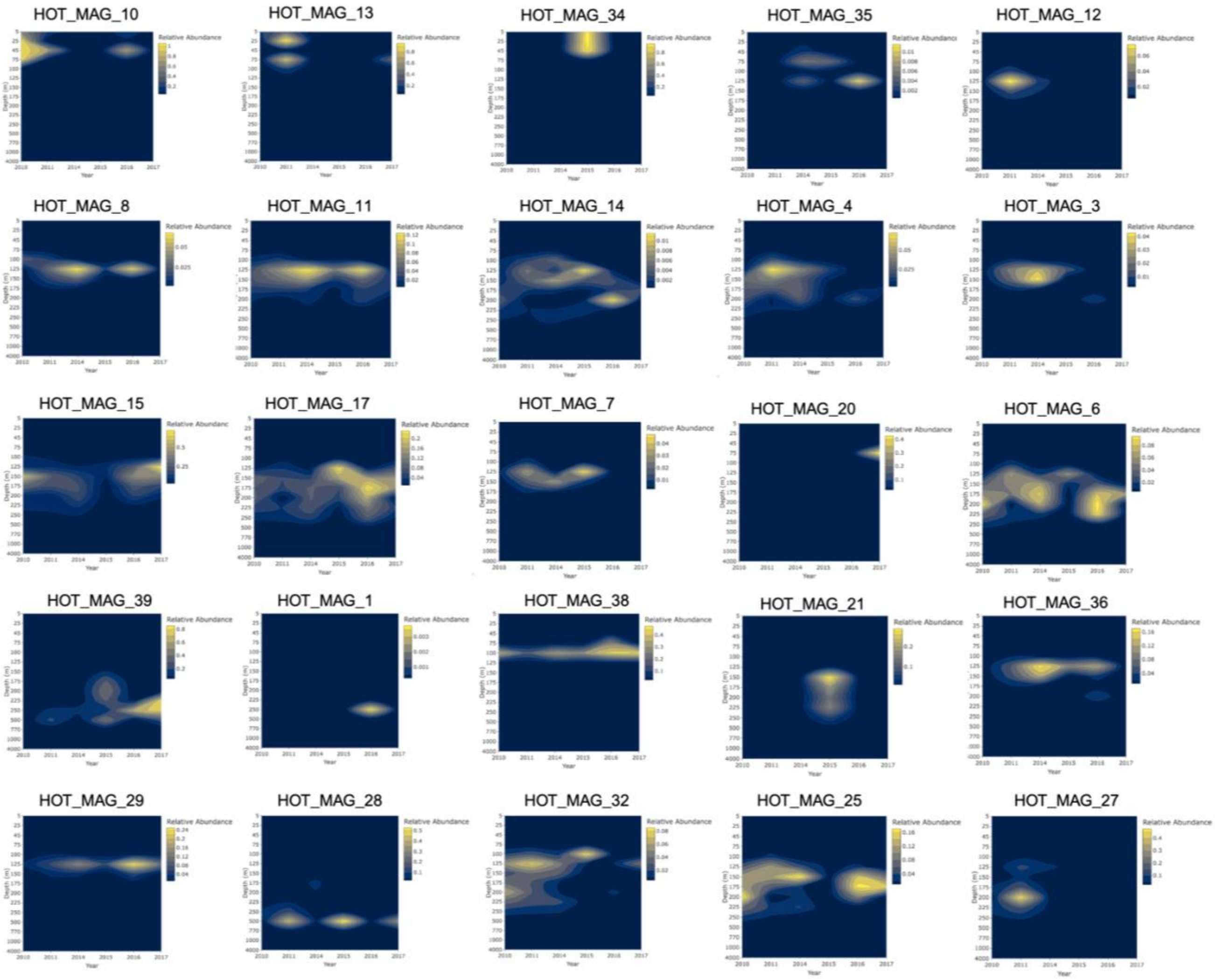

Algavirales

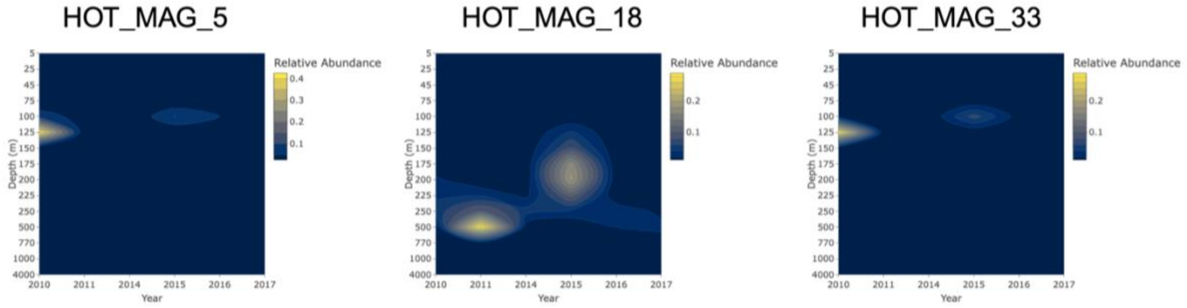

Fig S5: Spatiotemporal distribution of Imitevirales and Algavirales over a period of six years across 15 depths. HOT MAG represents the GVMAGs generated in this study

Data preprocessing and assembly

The quality of the raw reads was screened using FastQC [1] and MultiQC [2]. Next, the raw reads underwent trimming to eliminate adapter sequences and low-quality bases by Trimmolatic v.0.39 using the parameter HEADCROP:5 TRAILING:3 SLIDINGWINDOW:4:20 MINLEN:50 [3]. For each metagenome, reads were assembled into contigs with the de novo assembler MegaHit v.1.2.9 (with parameters -min-contig-len 1500) [4]. The quality of the assembly was further evaluated by MetaQUAST v.5.0.2 [5].

Generation of NCLDV contig database

All contigs from each metagenome were filtered through Virsorter2 v.2.2.4 [6] with the parameter --include-groups "dsDNAphage,NCLDV,RNA,ssDNA,lavidaviridae" and contigs classified under the NCLDV group were extracted. Bowtie2 v.2.5.1 [7] and samtools [8] were used to identify reads mapping to initially classified NCLDVs contigs. From the total mapped reads, 183 million reads derived from the >0.2 μm size fraction were pooled for a single NCLDV-specific reassembly using MegaHit v.1.2.9 [4] (Li et al 2015). Reads from the >0.2 μm fraction were chosen because giant viruses are most frequently observed in the > 0.22 μm size fraction [9, 10]. All contigs from initial MegaHit assemblies and the NCLDVs-specific reassembly were clustered using cd-hit-est v.4.8.1 [11] at >95% ANI across the total sequence , and contigs over 10 kbp were selected to generate a database containing 244,464 nonredundant contigs (referred to as nonredundant >10kbp contigs database). A multi-step quality filtering on this nonredundant >10kbp contig database was performed to identify putative NCLDVs contigs. First, Virsorter2 v.2.2.4 [6] was used to initially filter contigs belonging to NCLDVs. Then, these contigs were analyzed through Viralrecall v.2.0 [12] to identify 1739 contigs that contain at least one NCLDV-specific marker gene in the Virsorter2-identified NCLDVs contigs. The NCLDVs marker genes include NCLDV major capsid protein (MCP), superfamily II helicase (SFII), virus-like transcription factor (VLTF3), B-family DNA polymerase (PolB), and A32-like ATPase (A32), DNA-dependant RNA polymerase alpha subunit (RNAPL), DNA-dependant RNA polymerase BETA subunit (RNAPS), transcription elongation factor II-S (TFIIS), family II topoisomerase (Topoll) [13]. A final step of quality filtration was performed using geNomad v.1.7.1 [14] to remove 243 contigs from other viral groups. These quality filtering steps resulted in 1496 nonredundant >10kb NCLDV contigs for further analysis.

Taxonomic identification of NCLDV contigs

The taxonomy of the NCLDV contigs was assigned based on seven key NCLDV marker proteins previously mentioned. Briefly, all protein sequences from the phylum *Nucleocytoviricota* (taxid: 2732007) were extracted from the NCBI database. The marker proteins identified in the NCLDV

contigs were then subjected to BLASTp [15] against the *Nucleocytoviricota* protein sequences using the *E*-value of 1×10^{-5}, percent identity of >60% and query coverage of >50%.

Relative abundance of NCLDV contigs in water column

To identify the abundance of NCLDV contigs across the water column, reads from >0.2 µm fraction samples of both ALOHA 1.0 and ALOHA 2.0 datasets were mapped onto 1496 nonredundant >10kb NCLDV contigs using bowtie2 v.2.5.1 with default parameters [7]. Anvi'o v.8 (Eren et al 2015) was used for the calculation of the Q2Q3 mean coverage for each sample, which yields a more accurate coverage value by reducing overestimation of coverages from reads mapping to conserved regions and underestimation from hypervariable regions. Contigs with >0 Q2Q3 coverage were considered to be present in samples from different depths. The Q2Q3 coverage of each contig was further normalized to the max of that sample.

Relative abundance of NCLDV contigs in abyssal ocean

In order to investigate the abundance of NCLDVs in the abyssal ocean, we analyzed 63 previously collected metagenomic samples obtained over a period of three years from 2014 to 2016 in deep-moored sediment traps at 4000m at the Station Aloha in the North Pacific Subtropical Gyre (Poff et al. 2020, Luo et al 2022). Reads from the sediment trap dataset were mapped onto the nonredundant >10kb NCLDV contigs generated in the present study using BWA v.0.7.17 (Heng 2009) with default parameters. The Q2Q3 mean coverage for each sample was calculated using Anvi'o v.8 [16]. Similar to the planktonic samples, contigs with a non-zero Q2Q3 coverage were considered to be present in the sediment trap dataset and the Q2Q3 coverage value of a contig was normalized to the max of that sample. NCLDV contigs showing significant Pearson's correlation ($p < 0.05$) with log-transformed particulate carbon flux were identified using the WGCNA package in R [17], with the parameters specified by [18].

Identification and depth distribution of auxiliary metabolic genes

Auxiliary metabolic genes were identified using the Kyoto Encyclopedia of Genes and Genomes (KEGG) [19]. Class I AMGs were defined as genes present in KEGG metabolic pathways, while Class II AMGs were annotated with general metabolic functions or absent from metabolic pathways, presumably performing peripheral roles such as nutrient transport [20]. Giant virus-encoded AMGs were identified using MetaCerberus v.1.3.1 [21] by annotating against KEGG v.Jan24 (Kanehisha 2000) with the E-value of 1×10^{-5}. NCLDV contigs containing these AMGs were further identified. To examine the depth distribution patterns of these AMGs-encoding NCLDV contigs, we used the normalized Q2Q3 values of the contigs across various depths.

Generation of giant virus metagenome-assembled genomes

Nonredundant >10 kbp contigs database (nonredundant >10 kb contigs from the initial MegaHit assemblies and the NCLDV-specific reassembly) were subsequently binned using MetaBat2 v.2.16 [22]. The program MetaBat2 v.2.16 was utilized to bin the contigs, since this program considers sequence coverage and tetranucleotide frequencies which are consistent in viral genomes. MetaBat2 uses differential coverage to bin the contigs coming from different sample. The parameters -s 100000, -m 10000, --minS 75, and --maxEdges 75 were employed for binning, as these more stringent parameters are expected to produce more conservative and high-confidence binning results compared to the default parameters [23]. All the bins were further screened through Viralrecall v.2.0 [12] in order to identify ones corresponding to the putative NCLDV genome. The bins are considered as NCLDV if it contains at least 4 out of 5 highly conserved, NCLDV-specific protein families (A32, PolB, VLTF3, MCP and SFII) and had a total size of >100 kbp. In addition, contigs with a ViralRecall score < 0 were manually eliminated from the bins, as these contigs are potentially from cellular origin. The initial GVMAGs were further analyzed through TIGTOG [24] to remove 2 genomes with unusually large size and/or low coding density, indicating non-viral genomes. Overall, the use of these strict binning parameters, along with excluding potential cellular and bacteriophage contigs, allowed for the recovery of 37 high-quality GVMAGs.

Phylogenetic construction of the NCLDV MAGs

In order to assess the phylogeny of the generated NCLDV MAGs, proteins encoded by these MAGs were predicted using Prodigal v. 2.6.3 [25]. A concatenated alignment was created using 37 NCLDV MAGs and 1383 reference giant virus genomes from a previously compiled giant virus database [26]. This alignment was generated with the program ncldv_markersearch [13] and utilized the NCLDV PolB marker gene. The alignment was trimmed using trimAI v.1.4.1 (parameter -gt 0.1) [27]. A maximum-likelihood phylogenetic tree was developed by IQ-TREE v.2.3.0 [28] with 1000 ultrafast bootstraps (model: Q.pfam+F+I+R10) and the tree was finally visualized using interactive tree of life (iTOL) [7, 29].

Spatiotemporal distribution of GVMAGs

The spatiotemporal distribution of 37 GVMAGs at Station ALOHA was assessed by mapping reads from >0.2 µm size fraction from both the ALOHA 1.0 and ALOHA 2.0 datasets using Bowtie2 v.2.5.1 [7]. This analysis covered depths ranging from 5 meters to 4000 meters over a total span of six years between 2010 - 2017. After the read recruitments, Q2Q3 mean coverage of each genome was calculated using Anvi'o v.8 [16] and the coverage value was normalized to a scale of 1. Finally, R-based tools (https://www.r-project.org/) were used to visualise NCLDV distributions.

Protein functional prediction and metabolic pathway analysis

All the predicted protein from each genome was annotated with MetaCerberus v.1.3.1 [21] against Pfam v.36 [30], VOG database v.80 [31], TIGRfam v.15 [32] and KEGG v.Jan24 [19]. Predicted proteins were manually inspected in order to identify genes associated with different functions of interest including central carbon metabolism, amino acid metabolism, DNA processing, etc. The tool MetaCerberus v.1.3.1 assigns KEGG Orthology (KO) number to predicted proteins from different giant virus MAGs. Finally, the KEGG pathway mapper [33] was used to generate the metabolic map associated with the assigned KO numbers.

References

1. Babraham Bioinformatics - FastQC A Quality Control tool for High Throughput Sequence Data. https://www.bioinformatics.babraham.ac.uk/projects/fastqc/. Accessed 15 Jan 2025.
2. Ewels P, Magnusson M, Lundin S, Käller M. MultiQC: summarize analysis results for multiple tools and samples in a single report. *Bioinformatics* 2016; 32: 3047–3048.
3. Bolger AM, Lohse M, Usadel B. Trimmomatic: a flexible trimmer for Illumina sequence data. *Bioinformatics* 2014; 30: 2114.
4. Li D, Liu CM, Luo R, Sadakane K, Lam TW. MEGAHIT: an ultra-fast single-node solution for large and complex metagenomics assembly via succinct de Bruijn graph. *Bioinformatics* 2015; 31: 1674–1676.
5. Mikheenko A, Saveliev V, Gurevich A. MetaQUAST: evaluation of metagenome assemblies. *Bioinformatics* 2016; 32: 1088–1090.
6. Guo J, Bolduc B, Zayed AA, Varsani A, Dominguez-Huerta G, Delmont TO, et al. VirSorter2: a multi-classifier, expert-guided approach to detect diverse DNA and RNA viruses. *Microbiome* 2021; 9: 1–13.
7. Langmead B, Salzberg SL. Fast gapped-read alignment with Bowtie 2. *Nat Methods* 2012; 9: 357.
8. Arumugam M, Harrington ED, Foerstner KU, Raes J, Bork P. SmashCommunity: a metagenomic annotation and analysis tool. *Bioinformatics* 2010; 26: 2977–2978.
9. Farzad R, Ha AD, Aylward FO. Diversity and genomics of giant viruses in the North Pacific Subtropical Gyre. *Front Microbiol* 2022; 13: 1021923.
10. Endo H, Blanc-Mathieu R, Li Y, Salazar G, Henry N, Labadie K, et al. Biogeography of marine giant viruses reveals their interplay with eukaryotes and ecological functions. *Nat Ecol Evol* 2020; 4: 1639–1649.
11. Li W, Godzik A. Cd-hit: a fast program for clustering and comparing large sets of protein or nucleotide sequences. *Bioinformatics* 2006; 22: 1658–1659.
12. Aylward FO, Moniruzzaman M. ViralRecall—A Flexible Command-Line Tool for the Detection of Giant Virus Signatures in 'Omic Data. *Viruses* 2021; 13: 150.

13. Moniruzzaman M, Martinez-Gutierrez CA, Weinheimer AR, Aylward FO. Dynamic genome evolution and complex virocell metabolism of globally-distributed giant viruses. *Nature Communications 2020 11:1* 2020; 11: 1–11.
14. Camargo AP, Roux S, Schulz F, Babinski M, Xu Y, Hu B, et al. Identification of mobile genetic elements with geNomad. *Nature Biotechnology 2023 42:8* 2023; 42: 1303–1312.
15. Altschul SF, Gish W, Miller W, Myers EW, Lipman DJ. Basic local alignment search tool. *J Mol Biol* 1990; 215: 403–410.
16. Eren AM, Esen OC, Quince C, Vineis JH, Morrison HG, Sogin ML, et al. Anvi'o: An advanced analysis and visualization platformfor 'omics data. *PeerJ* 2015; 2015: e1319.
17. Langfelder P, Horvath S. WGCNA: An R package for weighted correlation network analysis. *BMC Bioinformatics* 2008; 9: 1–13.
18. Ha AD, Moniruzzaman M, Aylward FO. High Transcriptional Activity and Diverse Functional Repertoires of Hundreds of Giant Viruses in a Coastal Marine System. *mSystems* 2021; 6.
19. Kanehisa M, Goto S. KEGG: Kyoto Encyclopedia of Genes and Genomes. *Nucleic Acids Res* 2000; 28: 27–30.
20. Hurwitz BL, Brum JR, Sullivan MB. Depth-stratified functional and taxonomic niche specialization in the 'core' and 'flexible' Pacific Ocean Virome. *ISME J* 2015; 9: 472–484.
21. Figueroa JL, Dhungel E, Bellanger M, Brouwer CR, White RA. MetaCerberus: distributed highly parallelized HMM-based processing for robust functional annotation across the tree of life. *Bioinformatics* 2024; 40.
22. Kang DD, Li F, Kirton E, Thomas A, Egan R, An H, et al. MetaBAT 2: An adaptive binning algorithm for robust and efficient genome reconstruction from metagenome assemblies. *PeerJ* 2019; 2019: e7359.
23. Moniruzzaman M, Martinez-Gutierrez CA, Weinheimer AR, Aylward FO. Dynamic genome evolution and complex virocell metabolism of globally-distributed giant viruses. *Nature Communications 2020 11:1* 2020; 11: 1–11.
24. Ha AD, Aylward FO. Automated classification of giant virus genomes using a random forest model built on trademark protein families. *npj Viruses 2024 2:1* 2024; 2: 1–9.
25. Hyatt D, Chen GL, LoCascio PF, Land ML, Larimer FW, Hauser LJ. Prodigal: Prokaryotic gene recognition and translation initiation site identification. *BMC Bioinformatics* 2010; 11: 1–11.
26. Aylward FO, Moniruzzaman M, Ha AD, Koonin E V. A phylogenomic framework for charting the diversity and evolution of giant viruses. *PLoS Biol* 2021; 19: e3001430.
27. Capella-Gutiérrez S, Silla-Martínez JM, Gabaldón T. trimAl: a tool for automated alignment trimming in large-scale phylogenetic analyses. *Bioinformatics* 2009; 25: 1972.
28. Nguyen LT, Schmidt HA, Von Haeseler A, Minh BQ. IQ-TREE: A Fast and Effective Stochastic Algorithm for Estimating Maximum-Likelihood Phylogenies. *Mol Biol Evol* 2014; 32: 268.

29. Letunic I, Bork P. Interactive Tree Of Life (iTOL) v5: an online tool for phylogenetic tree display and annotation. *Nucleic Acids Res* 2021; 49: W293–W296.
30. Mistry J, Chuguransky S, Williams L, Qureshi M, Salazar GA, Sonnhammer ELL, et al. Pfam: The protein families database in 2021. *Nucleic Acids Res* 2021; 49: D412–D419.
31. Trgovec-Greif L, Hellinger HJ, Mainguy J, Pfundner A, Frishman D, Kiening M, et al. VOGDB—Database of Virus Orthologous Groups. *Viruses* 2024; 16.
32. Haft DH, Selengut JD, White O. The TIGRFAMs database of protein families. *Nucleic Acids Res* 2003; 31: 371.
33. Kanehisa M, Sato Y. KEGG Mapper for inferring cellular functions from protein sequences. *Protein Science* 2020; 29: 28–35.
